# Can expressed prostatic secretions affect prostate biopsy decision of urologist?

**DOI:** 10.1590/S1677-5538.IBJU.2018.0292

**Published:** 2019-04-01

**Authors:** Osman Ergün, Erdem Çapar, Yunus Emre Göğer, Ayşe Gül Ergün

**Affiliations:** 1 Department of Urology, Medical Faculty, Süleyman Demirel University, Isparta, Turkey; 2 Department of Urology, Gediz State Hospital, Gediz,Turkey; 3 Department of Urology, Medical Faculty, Necmettin Erbakan University, Konya, Turkey; 4 Department of Microbiology, Isparta City Hospital, Isparta, Turkey

**Keywords:** Prostatitis, Prostate, Inflammation

## Abstract

**Objectives:**

To evaluate the frequency of NIH category IV prostatitis, and the use of expressed prostatic secretions tests in an effort to improve the reliability of prostate specific antigen as an indicator, to avoid unnecessary prostate biopsy.

**Materials and Methods:**

178 expressed prostatic secretion positive patients with serum prostate specific antigen levels of ≥ 2.5 ng / mL were included in present prospective study. The diagnostic evaluation included detailed history and physical examination, digital rectal examination, urine analysis, urine culture, and expressed prostatic secretions tests. Transrectal ultrasonography was used both to measure prostate volume and conduct 12 core prostate biopsy.

**Results:**

The prevalence of NIH category IV prostatitis was 36.9% (178 / 482) in our population of men. In our study patients (n: 178) prostate biopsy results were classified as; 66 prostatitis, 81 BPH, and 31 Pca. In asymptomatic prostatitis group, expressed prostatic secretion mean leucocyte ratio was higher compared to other two groups (p < 0.0001). The relation between number of expressed prostatic secretion leucocytes and prostatitis, benign prostate hyperplasia, and prostate cancer is analyzed. If 16 is taken as the cut of number for leucocyte presence, its sensitivity is 0.92 (AUC = 0.78 p = 0.01).

**Conclusions:**

The number of leucocytes in expressed prostatic secretion is higher in the chronic prostatitis group. If the leukocyte presence of 16 and above is taken as the cut off point, the sensitivity becomes 0.92 (AUC = 0.78). We firmly believe that our new cut off value may be used as to aid prostate specific antigen and derivates while giving biopsy decision.

## INTRODUCTION

In 1979 Wang et al. discovered Prostate Specific Antigen (PSA) (1). This antigen is secreted from the prostate ductal epithelial cells. PSA has become an important tool in Prostate cancer (Pca) screening and the incidence of Pca patients increased with the introduction of PSA into the clinical practice (1). PSA is organ specific but not cancer specific. However, increased PSA levels are also associated with conditions other than cancer, such as benign prostate hyperplasia (BPH), prostatitis and non-malignant conditions. These conditions can cause an increase in serum PSA levels that lead to potentially unnecessary biopsy procedures, increasing inconvenience for the patient, and causing over-diagnosis, over-treatment and elevated medical costs (2). Pca is determined in only 38% of biopsies performed on the basis of PSA elevation (3).

National Institute of Health (NIH) classified prostatitis into 4 categories in 1995. NIH defined Category IV as asymptomatic chronic prostatitis due to the presence of inflammatory cells either in expressed prostate secretions (EPS) or during histopathological examination of prostatic biopsies from asymptomatic men (4).

Empiric use of antibiotics in an asymptomatic patient in order to lower the PSA is common in current clinical practice. Previous studies examining this subject have yielded conflicting results. While some studies state that serum PSA can be reduced with a course of antibiotics (5, 6), other studies state that treating patients with antibiotics does not lower serum PSA levels (7-9). Some guidelines mentioned that “However, antimicrobial therapy for selected patients with category IV prostatitis associated with elevated PSA, infertility and those planned for prostate biopsy may warrant consideration (3: C)” (10). Generally in these studies, not only EPS positive patients, all clinically asymptomatic men with elevated PSA levels have evaluated.

There are several implications in the use of empiric antibitics for patients with elevated PSA levels such as cost and toxicity. In addition, there is concern that the indiscriminate use of empiric antibiotics could lead to the development of resistant bacterial species and thereby expose the patient to more resistant and aggressive sepsis.

Despite extensive research efforts, very few biomarkers of prostate cancer have been successfully implemented into clinical practice today and the PSA test is still the most important biomarker for the detection and follow-up of prostate cancer. Numerous studies of serum (PSA isoforms, prostate health index and other combinations), tissue (p63, AMACR, PSMA, Glutathione S-transferase P, etc.) and urine based (PCA 3, SPINK 1, Annexin A3, etc.) prostate cancer biomarker candidates have been presented during the last ten years (11-15). These biomarkers seem to be promising. Some of them is already in current daily practice and highly satisfactory results are being reported. However, these are not widely used because of cost, accessibility, their experimental manner or complexity of usage.

The aim of this prospective study was to further investigate the predictive role of the number of leukocytes in expressed prostate secretions in differentiating histological inflammation from PCa and other non neoplastic lesions. Also, to evaluate the use of EPS tests in an effort to improve the reliability of PSA as an indicator, to avoid unnecessary prostate biopsy.

## MATERIALS AND METHODS

After institutional review board (ethical committee) approval, all study participants provided informed written consent before enrolment and 482 men were recruited in the present prospective study. Patients underwent a four-specimen study according to Meares-Stamey method (16). After periurethral cleaning with alcohol sponge, the patient provided a VB 1 specimen consisting of the initial 5 to 10 mL of voided urine, followed by a VB 2 specimen. Patients were placed in a lithotomy position, and a physician wore liquid parafin-coated gloves to perform digital rectal examination and several bilateral and middle prostatic massages. After production of EPS by digital prostatic massage, the patient provided 5 to 10 mL of voided urine for the VB3 specimen. EPS was positive in 178 of 482 patients. Eligibility criteria included referral for initial PSA between 2.5 ng / mL and 20.0 ng / mL, EPS positivity and palpably normal digital rectal examination. Exclusion criteria were; 1) pyuria (more than 3 leukocytes), 2) urinary tract infection history 3) urethral disorder history, 4) 5 alpha reductase treatment 5) antibiotics or anti-inflammatory therapy within last 2 months, 6) neurological disorders with an impact on lower urinary tract function, 7) urethral stricture, 8) former prostate biopsies or genitourinary surgery, 9) urological intervention within last month, 10) presence of acute urinary retention, 11) permanent urethral catheter, and 12) former prostatitis diagnosis and antibiotic therapy history. After a detailed physical examination, a digital rectal examination was made. Urine analysis, urine culture, anti-biogram and EPS tests were done for all participants. Prostate fluid samples following prostate massage were obtained from all patients. The liquid samples were counted for leukocytes on microscope slides with a large enhancement (40 X), if the number of leukocytes was ≥ 10, it was considered as positive for prostate inflammation. Prostate volume measurements were made and Ultrasound-guided 12 core prostate biopsy was taken using 18 G 30 cm needle automatic prostate biopsy gun. Biopsy results were classified as BPH, NIH category IV prostatitis and Pca. If Pca and prostatitis observed together in biopsy pathology, it was classified in Pca group. If ≤ 3 of 12 core had evidence of prostatitis, it was classified in BPH group. If ≥ 4 of 12 core had evidence of prostatitis, it was classified in prostatitis group. Prostat volumes were measured using 0.5 (LxWxH) formula (L: length from top to bottom W: horizontal length H: anterior posterior length). Data analysis was made using SPSS 15.0 Software Package Program (descriptive statistical analysis, ANOVA post hoc test, correlation test, Roc curve).

## RESULTS

The prevalence of NIH category IV prostatitis was 36.9% (178 / 482) in our population of men. In our study patients (n: 178) prostate biopsy results were classified as; 66 prostatitis, 81 BPH, and 31 Pca (Table-1). None of the patients, diagnosed with prostatitis in histopathological analysis had symptomatic or asymptomatic prostatitis history or former antibiotics therapy due to prostatitis. There was no statistically significant difference between prostatitis, BPH, and Pca groups in terms of age (p = 0.37). Likewise no statistically significant difference was seen among the three groups in terms of prostate volume (p = 0.40). A statistically significant relation and a strong correlation between tPSA level and prostate volume was present (p < 0.05 r = 0.68).

**Table 1:** General features of the Patients and Distribution.

	Asymptomatic prostatitis	BPH	Prostate Ca	p
Age	54.50 ± 7.8	62.8 ± 8.8	64.3 ± 9.4	0.37
Total PSA (ng/mL)	8.31 ± 16.4	7.38 ± 3.9*	10.4 ± 8.5*	0.022*
Free PSA (ng/mL)	1.26 ± 1.13	1.15 ± 0.61	1.16 ± 0.50	>0,05
fPSA/tPSA	0.16±0.09	0.21±0.14+	0.11±0.09+	0.001+
Prostate vol. (mL)	40.38 ±29.4	41.6 ± 20.9	37.1 ± 21.7	0.40
EPS	26.7±9.7&	13.8±13.9	11.3 ± 14.2	<0.0001&

mean±SD

* Difference between PCa and BPH;

+ Difference between Pca and BPH;

& Difference prostatitis with Pca and BPH

When prostatitis, BPH, and Pca groups were evaluated according to tPSA levels, there was a statistically significant difference between BPH and Pca (p = 0.022). In terms of tPSA, there was no statistically significant difference between the BPH-prostatitis (p = 0.21) groups and Pca-prostatitis groups (p = 0.45) (p > 0.5). In terms of free PSA (fPSA), no statistically significant difference was seen between in the BPH-Prostatitis group (p = 0.99), and in BPH-Pca (p = 0.72), or Prostatitis-Pca (p = 0.84) groups.

**Figure 1:**
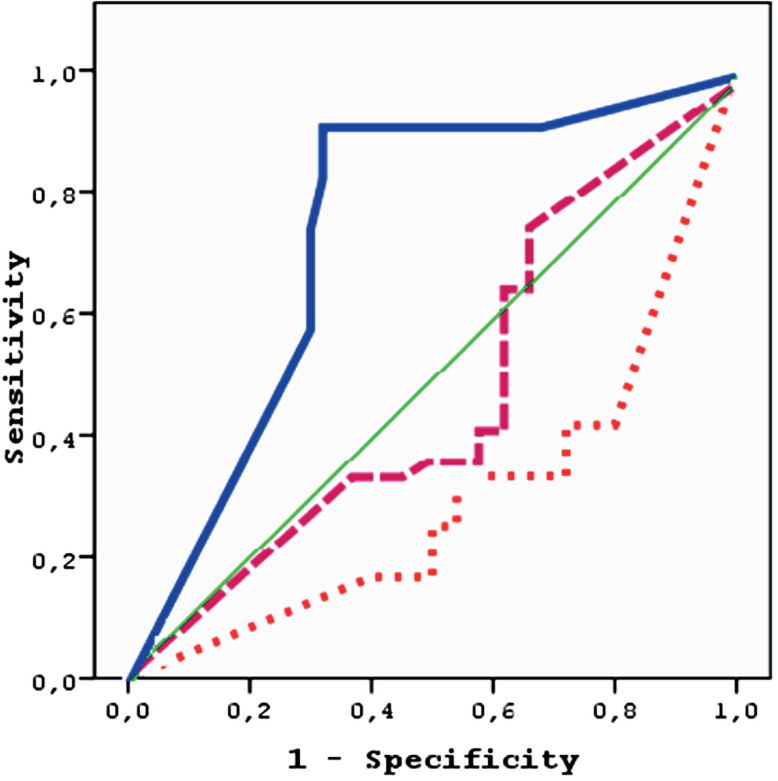
Number of Leukocytes in Prostate Massage Fluids; Prostatitis, BPH, and Pca Relations (Straight line: prostatitis, Dashed line: BPH, Dots: Pca).

In the prostatitis group the number of leukocytes in the EPS was higher than in the other two groups (p < 0.0001). The relation between the number of leukocytes in the EPS and prostatitis, BPH, and Pca was also evaluated. The most appropriate cut off value for the amount of leucocytes in the prostate massage fluid was determined as ≥ 395 (in a high enhancement area with a mean of ≥ 16) as shown in Figure-1. In the Roc curves drawn, statistical significance was only determined in the area under the curve between the number of leukocytes with prostatitis (AUC = 0.78 p = 0.01 prostatitis sensitivity: 0.92).

## DISCUSSION

The incidence of Pca patients who diagnosed at low stage, increased because of widespread using PSA testing. The rate of detecting Pca clinically in elevated levels of serum PSA ranges between 17.5% and 38% (3). PSA estimated sensitivity at the cutoff 4.0 ng / mL value is 21% for detecting any prostate cancer and specificity is 91% (17). There is however, a wide range (10-80%) of reported false positivity (18, 19). Determining the accuracy of PSA testing is confounded by the fact that most men with normal PSA values do not undergo biopsy, overestimating sensitivity and underestimating specificity.

Chronic prostatitis is a disease still without a clear etiology. Polymorphonuclear leukocyte invasion is determined in the intra-prostatic channels and peri-prostatic tissue during histopathological analyses (20, 21). Chronic prostatitis may present itself with very different clinical presentations. According to their clinical and laboratory findings, NIH classified prostatitis into 4 categories in 1995.

Various studies about the prevalence of prostatitis subtypes determined by NIH have been conducted. In studies made with needle biopsy analyses, prostatitis incidence ranging on a large scale between 17.2% and 42% have been reported (22, 23). However, studies on prevalence of NIH category IV prostatitis are very scarce. In a study made on a prostate cancer awareness screening program population, NIH category IV prostatitis incidence was determined as 32.2% (24). In the present study a rate of 36.9% was determined.

PSA, an important tumor marker, is organ but not cancer specific, studies about new methods verifying PSA results are in progress. To reduce unnecessary biopsies and to improve on PSA specificity, past research primarily investigated PSA derivatives. These have included PSA velocity, PSA density, age-specific PSA, fractionated PSA and percentage of free prostate-specific antigen (% fPSA) (25). Forexample; % fPSA is of some diagnostic use, although without significantly reducing the rate of negative biopsies. In particular, in a recent study, % fPSA has been documented as a poor discriminator between chronic prostatitis and Pca (11).

Urologists often manage asymptomatic men with a high serum PSA level by observation after antibiotic treatment. Because inflammation can often result in elevated PSA at documented chronic bacterial prostatitis patients. But studies examining this subject in the asymptomatic population have yielded conflicting results. While some studies state that serum PSA can be reduced with a course of antibiotics (5, 6), other studies state that treating patients with antibiotics does not lower serum PSA levels (7-9). Recently, in a prospective, controlled study by Greiman et al., 136 asymptomatic men with elevated PSA were divided into a ciprofloxacin treatment group (n = 63) or observation group (n = 73) where the study group received six weeks of ciprofloxacin and the observation group did not receive treatment (26). They continued routine follow-up for an average of 4.6 years with routine PSA, exam and prostate needle biopsy per clinical practice guidelines. The primary endpoint of this study was change in serum PSA, while the secondary endpoint was presence or absence of prostate cancer on biopsy. This study found a borderline statistically significant change in serum PSA between patients randomized to a 6-week course of fluoroquinolones versus observation, and no difference in positive prostate biopsy results. The authors suggest that patients with an elevated serum PSA could not treated with antibiotics in the absence of clinical symptoms of prostatitis. The literature does not support the evidence that antibiotics alter PSA levels except in the presence of bacterial prostatitis. Also, Heldwein et al. showed that PSA levels tend to fall when repeated after 45 days, regardless of antibiotic treatment (27).

Prostatitis should be diagnosed if there are 10 and more leukocytes present in a high enhancement area (40 X). According to the findings of the present study, number of polymorphous nuclear leucocytes in prostatitis group is higher than in BPH and Pca groups (p < 0.0001). Normally, 10 or more leucocyte counts are sufficient for prostatitis diagnosis. However, the number of leukocytes at the prostate massage fluid in the prostatitis group was higher than the other two groups, so that have us an idea.

We thought that, if a new cut off value for leucocyte presence in prostatic fluid is determined, the predictability of prostatitis will be increased and thus unnecessary biopsies may be avoided. The outcome of the Roc curve analysis revealed that average leucocyte presence of ≥ 16 or total leucocyte presence of ≥ 395 is most appropriate and should be taken as the new cut off point in a large enhancement area. The analyses revealed AUC = 0.78 and sensitivity for prostatitis as 0.92.

In summary, if the number of leucocytes is 16 and above in the prostatic massage fluid, this may most probably an indicator of prostatitis driven PSA increase. Previous to a biopsy decision, this new cut off value may be applied in clinical practice when encountering a asymptomatic patient presenting for the first time with an elevated PSA and without clinical evidence of prostatitis. Also, we recommend in these patients, it may be more beneficial to utilize other tools such as PSA velocity, PSA density, complexed PSA or newer clinical tools such as the prostate health index. Our new cut off value may be used to lend assistance these tools before biopsy decision.

Our study has some methodologic factors that might affect accuracy of our estimates. We didn't correlate the EPS results with histology results of our biopsy. Because, we classified chronic inflammation histologically as; available or unavailable. We didn't make detailed classification. Chronic inflammation of the prostate was defined as infiltration of prostate biopsy specimens by inflammatory cells, lymphocytes, plasma cells and / or histiocytes. Irani et al. classified inflammation with regard to the histological grade and aggressiveness of the inflammatory process: histological grades 0 and 1 were regarded as the “non-inflammation group”, and histological grades 2 and 3 as the “inflammation group” (28). This classification system is more objective for the correlation of EPS results with histology results. Engelhardt et al. used this classification for to evaluate the possible correlations between chronic asymptomatic inflammation of the prostate type IV and prostate cancer in patients undergoing radical prostatectomy (29). In another study, it was used for to investigate the association of the expression of tumor necrosis factor-α (TNF-α) with asymptomatic inflammatory prostatitis National Institutes of Health (NIH) category IV and prostatic calculi, in patients with obstructive benign prostatic hyperplasia (BPH) treated by transurethral electroresection of the prostate (TURP) (30).

## CONCLUSIONS

The number of leucocytes in EPS is higher in the chronic prostatitis group. If the leukocyte presence of 16 and above is taken as the cut off point, the sensitivity becomes 0.92 (AUC = 0.78). We firmly believe that our new cut off value may be used as to aid PSA and derivates while giving biopsy decision. Further investigations will be necessary to evaluate our new cut-off value accuracy and determine exact cut-off points.

## ABBREVIATIONS

PSA: = Prostate Specific Antigen
Pca: = Prostate cancer
BPH: = Benign prostate hyperplasia
NIH: = National Institute of Health
EPS: = Expressed prostatic secretions
tPSA: = Total PSA
fPSA: = free PSA
